# Deep and lasting response and acquired resistance to BRAFV600E targeting in a low-grade ovarian cancer patient

**DOI:** 10.1080/15384047.2023.2193116

**Published:** 2023-03-26

**Authors:** Sandro Anchisi, Anita Wolfer, Bettina Bisig, Edoardo Missiglia, Amine Tiab, Ehab Mohamed Kamel, Olivier Michielin, George Coukos, Krisztian Homicsko

**Affiliations:** a Oncology, Centre Hospitalier du Valais Romand, Sion, Switzerland; b Swiss Agency for Therapeutic Products, SwissMedic, Bern, Switzerland; c Institute of Pathology, Department of Laboratory Medicine and Pathology, Lausanne University Hospital and Lausanne University, Lausanne, Switzerland; d Service of Histocytopathology, Institut Central des Hôpitaux Valaisans, Sion, Switzerland; e Nuclear Medicine, Centre Hospitalier du Valais Romand, Sion, Switzerland; f Department of Oncology, Lausanne University Hospital, Lausanne, Switzerland; g Ludwig Institute for Cancer Research, Lausanne Branch, Lausanne, Switzerland; h Swiss Institute of Bioinformatics, Lausanne, Switzerland; i Agora Cancer Research Center, Lausanne, Switzerland

**Keywords:** Ovarian cancer, BRAF, resistance, adaptation

## Abstract

The treatment of BRAFV600E mutant melanoma has been revolutionized by BRAF inhibitors. Furthermore, the BRAF/MEK combination has shown further improvement in clinical outcomes in advanced and in adjuvant melanoma patients. In low-grade ovarian tumors, BRAF inhibitor use has been also proposed. Here we present a patient with an excellent, lasting response to BRAF therapy alone. At first progression, after more than two years on BRAF monotherapy, we could not identify any molecular mechanisms explaining resistance. After a switch to dual BRAF/MEK therapy, the patient responded. However, despite the initial response clinical the patient again progressed, this time with the appearance of a KRAS G12C mutation, which could not be overcome by BRAF/MEK therapy. We provide evidence that BRAF inhibitor alone can be highly beneficial in BRAF mutant low-grade ovarian tumors and the resistance mechanisms are similar to that of other BRAF mutant tumors, including in melanoma.

## Main text

**Figure 1.**
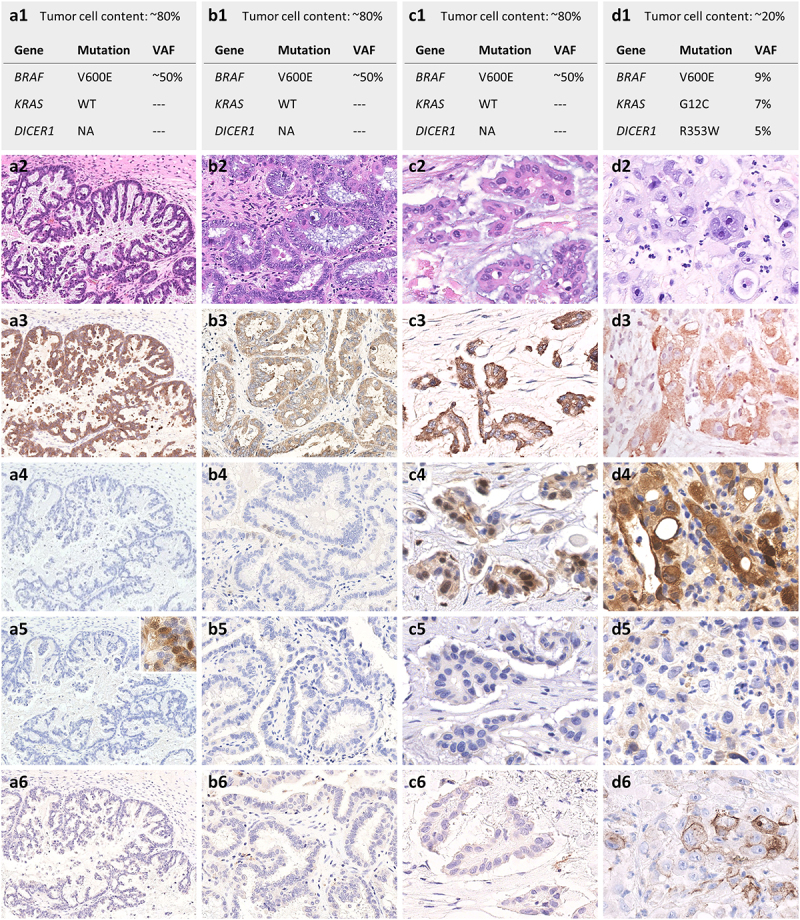
Summary of molecular and histopathological findings from initial diagnosis in 1997 (column A), through peritoneal relapses in 2006 (B) and 2013 (C), to hepatic progression in 2017 (D). A1-D1, molecular features. *BRAF* V600E mutation was detectable from initial diagnosis (A1) and throughout all subsequent samples (B1-D1), while *KRAS* gene, initially wild type (WT), acquired a G12C mutation at last progression under dual BRAF/MEK inhibition (D1). An additional *DICER1* R353W variant of uncertain functional significance was identified in the last sample (D1). *DICER1* mutation data of previous samples are not available (NA). VAF, variant allele frequency. A2-D2, histopathological aspects. From a low-grade serous carcinoma comprising initially an extensive borderline tumor component (A2, Hematoxylin, and eosin (HE), original magnification 100x; B2, 200x; C2, 400x), the neoplastic proliferation progressed into a high-grade, poorly differentiated carcinoma (D2, HE, original magnification 400x). A3-D3, BRAF V600E immunostaining. In line with the detection of *BRAF* V600E mutation by sequencing (A1-D1), the mutant protein BRAF V600E was detected by immunohistochemistry in all sequential samples, including in the borderline tumor component (A3-D3, clone VE1, original magnification 100x, 200x, 400x, 400x). A4-D4, pERK immunostaining. Tumor samples were mostly negative for pERK in 1997 and 2006 (A4-B4, original magnification 100x and 200x). Focal nuclear and cytoplasmic expression was detected in 2013 (C4, 400x), while more diffuse staining (strong in the cytoplasm and weaker in the nuclei) was observed in 2017 (D4, 400x). A5-D5, pAKT immunostaining. No significant expression of pAKT was identified in any of the tumor samples (A5-D5, original magnification 100x, 200x, 400x, 400x; A5 inset: external positive control for pAKT, 400x). A6-D6, PD-L1 immunostaining. While there was no tumor cell staining for PD-L1 in the initial sample and peritoneal relapses (A6-C6, clone SP263, original magnification 100x, 200x, 400x), membrane expression of this protein became detectable in 30% of the tumor cells in the liver metastasis (D6, 400x).

We fortuitously diagnosed a 42-year-old woman in January 1997 with a low-grade serous cystadenocarcinoma of the ovary during surgical sterilization. She presented with bilateral ovarian masses, numerous peritoneal implants, and infiltration of 2 out of 6 right iliac lymph nodes, pathological stage of pT3c pN1, FIGO IIIC, with no clinical evidence of distant metastasis. A total hysterectomy with bilateral salpingo-oophorectomy and omentectomy was performed (showing an extensive serous borderline tumor component, Figure 1A), with no visible residual disease, followed by six cycles of adjuvant chemotherapy with paclitaxel and carboplatin.

**Figure 2.**
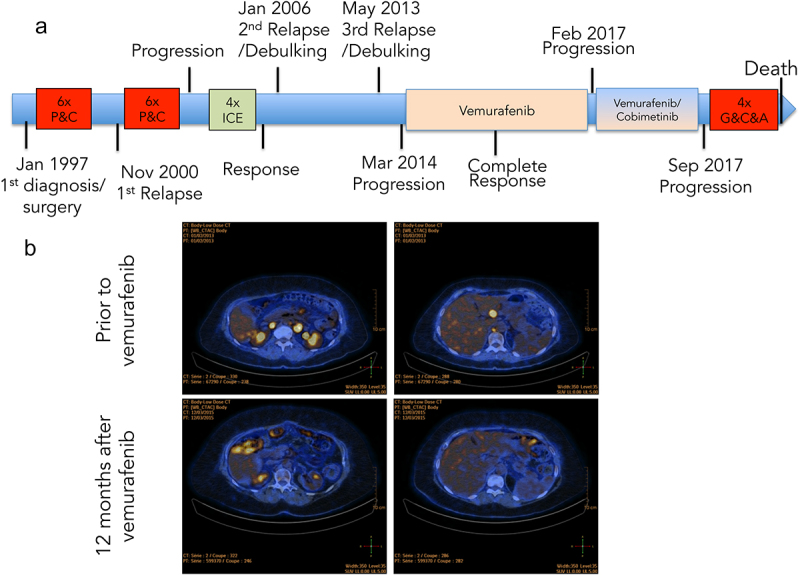
Clinical and radiological description of the case of LG-SC.

Four years later (Figure 2A), a biochemical recurrence prompted a CT scan, which showed retroperitoneal lymphadenopathies and pelvic and perihepatic hypodense masses. At laparotomy, only a biopsy was performed due to the extent of the peritoneal carcinomatosis. After six cycles of paclitaxel and carboplatin, CA-125 further increased, and chemotherapy was changed to 4 cycles of Ifosfamide, etoposide, and carboplatin. A partial response with residual radiological disease and normalization of the CA-125 level (30 kU/l) was obtained. The patient was followed clinically until January 2006, when CA-125 increased again with symptomatic disease, and a second partial debulking surgery was performed. Histopathological analysis showed a borderline serous tumor with rare foci of low-grade serous carcinoma (Figure 1B2). In light of the histopathological diagnosis, no further treatment was delivered. Seven years later, in May 2013, the patient underwent a partial debulking surgery to relieve recurring, partial bowel obstruction. Microscopic examination confirmed the diagnosis of relapsing low-grade serous carcinoma (Figure 1C2). A few areas showed more marked nuclear pleomorphism and foci of necrosis, suggesting a high-grade component. Molecular analysis, performed by classical pyrosequencing, identified a V600E mutation in *BRAF* (exon 15) and no mutation in exon 2 of *KRAS* (Figure 1C1). Twelve months later, she complained of nausea with vomiting due to progressive peritoneal carcinomatosis (PET-CT scan 06.03.2014, CA-125: 302 kU/l). Given the presence of the *BRAF* V600E mutation, treatment with the mutant BRAF inhibitor, vemurafenib, was proposed at a dose of 480 mg bid. Due to grade 3 side effects, the dosing was adapted to 240 mg bid.

A, The clinical course of the ovarian cancer patient and treatments B, Two representative images per time point; upper panels: before initiation of vemurafenib, lower panels: 12 months after therapy.

Eight months after the introduction of vemurafenib, a CT scan (Figure 2B) showed the disappearance of all visible lesions, and the CA-125 level had normalized to 27 kU/l. After 12 months, a PET scan (12.03.2015) confirmed the complete radiological response. The patient remained with a complete response for three years. In February 2017, we detected progression of the peritoneal disease with a single, new liver metastasis. After our molecular tumor board discussion, the MEK-inhibitor cobimetinib was added to vemurafenib. The combination therapy again resulted in tumor response, evident already after two months of treatment. The partial response lasted for seven months when the patient showed further progressive peritoneal carcinomatosis and numerous liver metastases. A new liver biopsy showed infiltration by a poorly differentiated carcinoma, composed of highly pleomorphic tumor cells admixed with multiple inflammatory cells and alternating with foci of necrosis (Figure 1D2). By immunohistochemistry^1^, the tumor cells were positive for cytokeratin 7, WT1, GATA3 and BRAF V600E mutant protein (Figure 1D3); they were negative for PAX8, estrogen and progesterone receptors and HER2. Overall, the clinicopathological picture was consistent with a metastatic progression of the ovarian carcinoma. Starting from this biopsy and matched constitutional DNA, we performed a comprehensive molecular tumor profiling using an in-house developed next-generation sequencing (NGS) panel, covering the complete coding sequences of 400 cancer-associated genes, and the patient was discussed at our molecular tumor board. This analysis showed the persistence of the *BRAF* V600E mutation and the appearance of a novel *KRAS* G12C mutation that was absent in the initial tumor. The allele frequencies of the two mutations were similar (Figure 1D1). Also, a somatic mutation of unknown functional significance was identified in the RNA endoribonuclease, *DICER*. No mutation was detected in TP53. Copy number variation (CNV) analysis revealed very few chromosomal abnormalities (21p low copy number gain and 22q deletion).

To understand the *KRAS* mutation’s kinetics, we sequenced the relevant hotspot regions in the available sequential samples using pyrosequencing. The *BRAF* V600E mutation had been present since the initial diagnosis^2^, including in the borderline tumor ^3^, while the *KRAS* G12C mutation became detectable only in the last liver metastasis (Figure 1,A1-1D1, and 1A3-1D3). We also looked at changes in the activities of the MAPK and PI3K pathways in the same series of samples by staining for p-ERK and p-AKT, respectively. We found an increasing intensity of p-ERK staining parallel with disease progression (Figure 1A4-1D4). At the same time, AKT phosphorylation was absent in all tumors, a finding that corroborates the lack of mutations in the PI3K-mTOR pathway (Figure 1A5-1D5).

Furthermore, we found PD-L1 expression in 30% of the tumor cells in the progressing liver metastasis, while there was no detectable PD-L1 expression in the neoplastic component in any previous samples (Figure 1A6-1D6).

In the absence of a molecular target and in light of the long chemotherapy-free interval, we re-introduce a platinum-containing regimen with carboplatin and gemcitabine. A new CT scan after four cycles showed a massive progression, and the patient passed away shortly after.

## Discussion

Low-grade serous ovarian carcinoma (LG-SC) is a challenging disease to treat. While progression is slower than in high-grade serous carcinomas (HG-SCs), virtually all LG-SCs are resistant to chemotherapies. The clinical relevance of a 2-tier system to classify serous ovarian carcinomas (LG-SC *versus* HG-SC),^4,5^ demonstrated a significantly longer PFS for LG-SCs and a higher risk of death for HG-SCs^6^.

This survival difference reflects the divergent molecular pathogenesis of LG-SCs and HG-SCs^7^. The former has a low mutation burden with more stable genomes than high-grade tumors. The most frequent mutations in LG-SCs are *KRAS* and *BRAF* mutations^8^, which were not identified in HG-SCs^9^. *BRAF* and *KRAS* mutations are mutually exclusive in LG-SCs and their precursor lesions, borderline serous tumors (SBLTs)^10^. In line with the mutual exclusivity of *BRAF* and *KRAS* mutations, we only detected the *BRAF* V600E mutation in the analysis of the initial tumor. A higher frequency of *BRAF* V600E mutations in SBLTs was reported than in LG-SCs (71% *versus* 14%), and this alteration seems to be a marker of good prognosis. The clinical picture of our patient was unusual, given her advanced stage at presentation and the need for initial chemotherapy.

The *BRAF* V600E mutation is not only prognostic but also predictive. Vemurafenib and dabrafenib are oral agents, inhibiting mutant BRAF with proven efficacy in metastatic melanoma^11^. In a literature search, we found only two previous reports of ovarian cancer patients responding to a BRAF inhibitor monotherapy. One patient, treated within a phase I trial with dabrafenib, achieved stable disease as the best response^12^, while another reported a partial response lasting for 12,9 months^13^. Despite the reduced dosing, our patient achieved radiological and clinical complete response for three years. A prolonged complete response can also be obtained in a small subset of melanoma patients, but virtually all patients relapse, similar to our patient. The dual inhibition of BRAF and MEK had become the standard of care for BRAF V600 mutant melanoma in 2015^14^. Although sequential therapy, that is, treatment with a BRAF inhibitor followed by BRAF/MEK dual inhibition is not recommended in the management of *BRAF*-mutant melanoma, we decided to add the MEK inhibitor cobimetinib to vemurafenib. The addition of cobimetinib resulted in a novel, deep tumor response for seven months. The shortened time to progression probably reflects the heightened selection of preexisting BRAF inhibitor-resistant clones, which are also resistant to the BRAF/MEK combination. Comprehensive molecular profiling by NGS of the tumor progressing on BRAF/MEK inhibitors showed the appearance of a *KRAS* G12C mutation, which is typically mutually exclusive with *BRAF* mutations in LG-SCs. Notably, the similar allele frequencies of the *BRAF* and *KRAS* mutations suggested their co-existence in the same cancer cells and not a clonal evolution of tumor cells exclusively with *KRAS* mutation. In the case of melanoma, mutations in another *RAS* gene, *NRAS*, have been reported as one of the resistance mechanisms to BRAF inhibition^15^. We sought to define if *KRAS* mutant clones were already present in the primary tumor or any of the successive available tumor samples but could not identify the preexistence of *KRAS* mutation. This result is suggestive of an adaptive resistance mechanism. Mutations in the PI3K pathway are also frequent mechanisms of adaptation in melanoma in response to the BRAF/MEK inhibition^16^. However, we did not find any mutations in the PI3K pathway nor detected increased p-Akt staining.

This case illustrates the benefit of targeting mutant *BRAF* in LG-SCs. We speculate that a dual BRAF/MEK inhibition, which was not available in 2013, would have had a more prolonged impact on the patient’s survival. The major limitation to targeted therapy of *BRAF* mutant tumors is the omnipresence of adaptive mechanisms at the genomic level, but also in re-programming of the tumor microenvironment^17^, which could also be reflected by the increased PD-L1 expression at the last progression. Further systematic studies of *BRAF* mutant LG-SCs treated with dual BRAF/MEK inhibitors should be performed to confirm our observations of this single patient.

## Data Availability

This case report includes next-generation sequencing and immune histochemistry data, which could be provided upon reasonable request.
